# We need to bring R0 < 1 to treat cancer too

**DOI:** 10.1186/s13073-021-00940-9

**Published:** 2021-07-26

**Authors:** Devabhaktuni Srikrishna, Kris Sachsenmeier

**Affiliations:** 1 Breakbio Corp., 63 Bovet Road, #418, San Mateo, CA 94402 USA; 2 Translational Medicine-Oncology, AstraZeneca, 35 Gatehouse Drive, Waltham, MA USA

## Abstract

If each cancer cell produces on average more than one cancer cell, we see a net growth of the tumors and metastases and vice versa. We review recent clinical results for microsatellite stable metastatic colorectal cancer (MSS-mCRC) suggesting immunotherapy combinations with personalized vaccines, checkpoint inhibitors, targeted therapies, multikinase inhibitors, chemotherapies, and radiation that simultaneously slow cancer cell growth rate and enhance T cell killing rate of cancer cells may in future synergize to control the disease.

Demonstration of checkpoint inhibition (for PD-1/PD-L1 and CTLA-4) to enable T cell killing of tumor cells resulted in the 2018 Nobel prize. Different factors that affect checkpoint inhibitor response include the total number of non-synonymous mutations (tumor mutation burden, TMB ≥ 10 mutations per Megabase), and microsatellite instability-high (MSI-H) status, which were both recently approved by the FDA as biomarkers to identify patients who will likely respond to anti-PD-1 immunotherapy [1]. Although detection of minimum thresholds of tumor PD-L1 expression was FDA approved as a companion diagnostic for anti-PD1 inhibitors, many patients with low to no detectable PD-L1 expression also experience durable clinical benefit. Better response to checkpoint inhibitors (CPI) was observed in tumors with higher density of CD8 T cells at the center and periphery of a tumor, whereas immune evasion through reduction or loss of HLA expression on tumor or metastases may reduce response to CPI.

Therapeutic cancer vaccines train a patient’s immune system to elicit killer T cells that are tailored to the patient’s cancerous tissue or metastases. In an early trial of personalized cancer vaccines (PCV), it was found that eight patients with melanoma are still alive 4 years later (NCT01970358). Their PCV were based on up to 20 patient-specific tumor mutations (neoantigens) determined by DNA sequencing of their tumor tissue (some patients also received anti-PD1 inhibitors to enhance cancer cell killing by the PCV elicited T cells). Until recently, PCVs achieved modest clinical benefit, causing Genentech researchers to observe that administration of many T cell cancer vaccines failed to correlate with clinical benefit [2]. Subsequently, clinical responses (NCT03548467) were observed with Vaccibody’s PCV including approximately 50% of metastatic patients’ refractory to CPI (in melanoma, lung, bladder, renal, and head and neck cancers). Tumor regressions in approximately 50% of patients with head and neck cancer were also observed with Moderna’s PCV with CPI (NCT03739931).

Metastatic colorectal cancer (mCRC) remains a large unmet need. With the exception of a small minority of mCRC patients with MSI-H or high-TMB (discussed above), immunotherapies have so far not worked for the remaining vast majority of mCRC patients with microsatellite stable tumors (MSS-mCRC) and for whom there is no approval of CPI. Almost 50,000 people die every year in the USA, from mCRC and many times that globally. Below we highlight several recent, striking clinical results in MSS-mCRC that suggest future immunotherapy combinations [3] involving PCV, CPI, targeted therapies, multikinase inhibitors, and radiation that simultaneously slow cancer cell growth rate and enhance T cell killing rate of cancer cells that may synergize to control the disease.

## Immunotherapies for metastatic colorectal cancer (mCRC)

In colon cancer, greater T cell infiltration into the tumor’s periphery and center predicts improved survival [4]. However, unlike in other tumor types, PCV that elicit anti-tumor T cells when combined with checkpoint inhibition have not worked as hoped in CRC: in Moderna’s clinical trial (NCT03739931), there were no responses reported in 17 patients and only one out of 14 patients responded in BioNTech’s clinical trial (NCT03289962). In contrast to other tissues of origin, the limited response rates in a preponderance of independent trials, while each in a small number of patients, begs the question: is there something unique about the biology of tumors and metastases in MSS-mCRC making them resistant to T cell-based treatments such as PCV and CPI?

## R0 > 1

Patient’s tumors grow when cancer cells multiply faster than the treatments they take (e.g., T cells elicited by a vaccine) can kill the cancer cells. If on average each cancer cell gives rise to more than one cancer cell, we observe a net growth of the tumors and metastases (aside from pseudo-progression). Mathematically, this is analogous to the basic reproductive rate being greater than one, as observed for COVID-19 (R0 > 1), where each infected person or cancer cell, by analogy, transmits the virus to or multiplies into more than one additional infected person or cancer cell on average. T cell killing of cancer cells is not instantaneous and can take multiple hours in vitro [5]. Conversely, to observe a clinical response from T cell therapies, we hypothesize that killing of cancer cells by T cells needs to exceed the basic reproductive rate of cancer cells and be sustained safely for long enough to yield clinical results (R0 well below 1).

## R0 < 1 without vaccines

What can be done to tip the balance toward the rate of T cell killing exceeding the rate of cancer cell growth and safely bring R0 < 1 in MSS-mCRC? Chemotherapies, multikinase inhibitors, targeted therapies, and radiotherapies that slow cancer growth or stimulate the immune response combined with CPI (without cancer vaccines) listed in Table 1 show early signs of clinical efficacy and safety. For T cell killing to exceed the rate of cancer cell growth resulting in a clinical response, they must overcome at least some of the numerous immune evasion pathways or biological barriers reflected in the heterogeneity of molecular subtypes in colorectal cancer [8].

**Table 1:** Recent combination immunotherapies with chemotherapeutics, targeted therapies, multikinase inhibitors, and radiotherapy exhibiting early signs of clinical efficacy and safety in MSS-mCRC

Category of treatment	Clinical trial or study
With CPI	Bevacizumab, capecitabine, and atezolizumab (anti-PD-L1) (NCT0287319) Bevacizumab and capecitabine, pembrolizumab (anti-PD-1) (NCT03396926) Lenvatinib and pembrolizumab (anti-PD1) (NCT03797326) Cetuximab, FOLFOX, and avelumab (anti-PD-L1) (NCT03174405)
Regorafenib with CPI	Regorafenib and nivolumab (anti-PD1) (NCT03406871, NCT03712943, NCT04126733). Regorafenib and avelumab (anti-PD-L1) (NCT03475953) The combination of regorafenib with a checkpoint inhibitor (anti-PD1 or anti-PD-L1) in phase 1/2 trials in Japan (NCT03406871), France (NCT03475953), and the USA (NCT03712943, NCT04126733) resulted in slightly higher percentage of patients who survive beyond 1 year and partial response or stable disease in approximately 40% to 90% of the patients depending on the trial.
Radiation with regorafenib, CPI, or as monotherapy	Stereotactic body radiation therapy (SBRT) with ipilimumab (anti-CTLA-4) and nivolumab (anti-PD1). This is a trial in refractory MSS-mCRC adding two checkpoint inhibitors to localized radiation therapy (NCT03104439) called stereotactic body radiation therapy or SBRT (often conceptualized as an “in-situ” vaccine that stimulates a T cell response to the irradiated tumor) resulted in modest disease control rates. It included one complete response which was unexpected with radiation treatment alone. SBRT with regorafenib. An mCRC patient with liver metastases taking regorafenib who also received SBRT experienced a durable progression-free survival over 3 years [6]. SBRT monotherapy. In another study, three mCRC patients also experienced complete responses to SBRT after several prior treatments [7].

For example, regorafenib, a TKI/multikinase inhibitor, is FDA approved for chemotherapy-refractory mCRC and marginally increases survival (NCT01103323)—a small percentage of patients survive beyond 1 year. Its exact mechanism of action is unclear, but in vitro, it has been shown to have antiangiogenic effects and also slow the growth of a large variety of colon cancer cell lines [9]. Improved efficacy of regorafenib observed when combined with checkpoint inhibitors (Table 1) remains to be verified in larger, randomized clinical trials, but in principle, regorafenib may enhance the rate of T cell killing of cancer cells by giving endogenous T cells a better chance to kill the cancer cells in mCRC.

## R0 < 1 with vaccines

Based on a few striking case studies, the addition of vaccines to drugs that slow cancer growth rates plus CPI has potential for clinical benefit. As reported by Cleveland Clinic in November, 2020, an MSS-mCRC patient (in NCT03547999) recovered with a first-line treatment of a non-personalized vaccine (for two antigens, CEA and MUC1), fluoropyrimidine-based chemotherapy, and CPI. The patient remained cancer-free 1 year later. The combination of bevacizumab and various forms of fluoropyrimidine (chemotherapy) is a standard treatment in mCRC used to slow down the cancer’s progression. In a trial of eleven MSS-mCRC patients reported by Mayo Clinic in June, 2020, another non-personalized cancer vaccine for seven antigens combined with fluoropyrimidine-based chemotherapy (and either bevacizumab or cetuximab) resulted in three objective tumor responses according to RECIST v1.1 criteria (NCT03391232). In a trial of fifteen MSS-mCRC patients, a CAR-T cell treatment with fluoropyrimidine-based chemotherapy resulted in two partial responses (NCT03692429). Together, these motivate the concept of a need for drugs that slow cancer growth rates in addition to T cell based immunotherapy in MSS-mCRC.

In MSS-mCRC patients, liver metastases correlate with poorer responses including in response to regorafenib (NCT03406871, NCT04126733) when combined with checkpoint inhibitors. Recently, researchers discovered liver metastases may be causing a systemic reduction of T cells throughout the body, and in preclinical models, this was reversible by radiation delivered to the liver [10]. A systemic reduction of T cells caused by liver metastases suggests efficacy of clinical trials with immunotherapies such as vaccines that elicit T cells may be enhanced by excluding patients with liver metastases. However, two of the three responses in MSS-mCRC patients on the Mayo Clinic vaccine trial (NCT03391232) actually had liver metastases as did the MSS-mCRC patient at Cleveland Clinic vaccine trial who recovered. These examples, while small in number, suggest that an appropriate vaccine to elicit greater numbers of anti-tumor T cells could, in principle, be effective in overcoming immune escape potentially caused by systemic reduction of T cells from liver metastases.

Another strategy to enhance the efficacy of PCV so that T cell killing of cancer cells outpaces cancer cell growth could be to vaccinate patients at earlier stages or when they have smaller numbers of cancer cells, i.e., with minimal residual disease burden, limited metastatic spread, or immediately after resection of large tumors. BioNTech is commencing such a trial (NCT04486378) in early stage CRC patients with minimal residual disease. In many patients, colorectal cancer has already metastasized by their initial diagnosis making it imperative to find treatments not only in earlier stages but in late-stage (metastatic) settings with significant disease burdens and high rate of cancer cell growth.

## Conclusions

Carefully choosing immunotherapy combinations with PCV may in future prove to be useful for enhancing T cell killing of tumors and metastases in MSS-mCRC patients. While limited to a small number of patients, clinical case studies and early clinical trials suggest that, in future, synergistic therapeutic combinations including drugs that can simultaneously slow the cancer cell growth rate (e.g., targeted therapies, multikinase inhibitors, chemotherapies) and enhance the T cell killing rate of cancer cells (e.g., from a PCV, CPI, radiation) may be used to treat MSS-mCRC. If the balance can be struck in favor of T cell killing of cancer cells over cancer cell reproduction, this may ensure that fewer than one cancer cell is created for every cancer cell killed, bringing the basic reproductive rate of cancer cells below 1 (R0 < 1) to treat and control the disease.
